# The comprehensive on-demand 3D bio-printing for composite reconstruction of mandibular defects

**DOI:** 10.1186/s40902-022-00361-7

**Published:** 2022-10-04

**Authors:** Han Ick Park, Jee-Ho Lee, Sang Jin Lee

**Affiliations:** 1 Department of Oral and Maxillofacial Surgery, Asan Medical Center, College of Medicine, University of Ulsan, Seoul, South Korea; 2 Wake Forest Institute for Regenerative Medicine, Wake Forest School of Medicine, Winston-Salem, North Carolina 27157 USA

**Keywords:** Mandibular defect, Reconstruction of the mandible, Osteocutaneous-vascularized free flap, 3D bio-printing technology, Integrated tissue, Organ printing

## Abstract

**Background:**

The mandible is a functional bio-organ that supports facial structures and helps mastication and speaking. Large mandible defects, generally greater than 6-cm segment loss, may require composite tissue reconstruction such as osteocutaneous-vascularized free flap which has a limitation of additional surgery and a functional morbidity at the donor site. A 3D bio-printing technology is recently developed to overcome the limitation in the composite reconstruction of the mandible using osteocutaneous-vascularized free flap.

**Review:**

Scaffold, cells, and bioactive molecules are essential for a 3D bio-printing. For mandibular reconstruction, materials in a 3D bio-printing require mechanical strength, resilience, and biocompatibility. Recently, an integrated tissue and organ printing system with multiple cartridges are designed and it is capable of printing polymers to reinforce the printed structure, such as hydrogel.

**Conclusion:**

For successful composite tissue reconstruction of the mandible, biologic considerations and components should be presented with a comprehensive on-demand online platform model of customized approaches.

## Introduction

The mandible is a functional bone that supports facial structures and implements mastication and speaking. Tumor, osteomyelitis, trauma, radiation therapy, congenital disease, and medication-related osteonecrosis can lead to the destruction of the mandible. Significant defects of the mandible can result in the loss of function and aesthetics in patients, which has significant implications for their quality of life [1, 2].

Various graft materials have been used in clinical practice such as the xenogeneic graft, allograft, and autogenous bone. Xenogeneic graft and allograft require additional chemical and thermal processes to reduce immunoreaction and risk of infection. They also lack cell viability and biocompatibility compared to the autogenous bone [3–6]. Additional surgery required for the donor site and a limited amount of graft material are still limiting the widespread utilization of autogenous bone graft, even though it has an excellent biocompatibility in terms of osteogenesis, angiogenesis, and less risk of infection at the recipient site. A large mandibular defect, more than 6 cm full segment loss, often requires a vascularized autogenous graft to achieve adequate reconstruction. Several options have been suggested to achieve the reconstruction of segmental defects using titanium plates or meshes, however, long-term results have not been very successful [1]. Moreover, large segmental defect wounds are often accompanied by loss of adjacent soft tissue such as skin, oral mucosa, and muscle, thereby requiring composite tissue reconstruction in most cases. So far, osteocutaneous-vascularized free flap (OCFF) has been the gold-standard treatment option for composite tissue reconstruction of the mandible [7]. However, an inevitable defect at the donor site can be associated with considerable complications and morbidity [8].

With the recent development of bio-printing and tissue engineering using the 3D bio-printing technology, an alternative treatment to OCFF is emerging. The 3D computer-aided design and computer-aided manufacturing (CAD-CAM) module help in designing precise bio-printing materials which are customized to suit individual patient anatomy [9, 10]. Especially when implemented on an online platform, the overall process of 3D bio-printing can be controlled by multidisciplinary members of the reconstructive team, starting from the case analysis to the clinical application. In this review, biological considerations and its components will be addressed and discussed in conjunction with some of the pre-existing studies of 3D bio-printing for composite tissue reconstruction of the mandible. Also, a comprehensive on-demand online platform model will be suggested to facilitate customized approaches for individual patients.

## Functional anatomy for composite tissue reconstruction

**Fig. 1:**
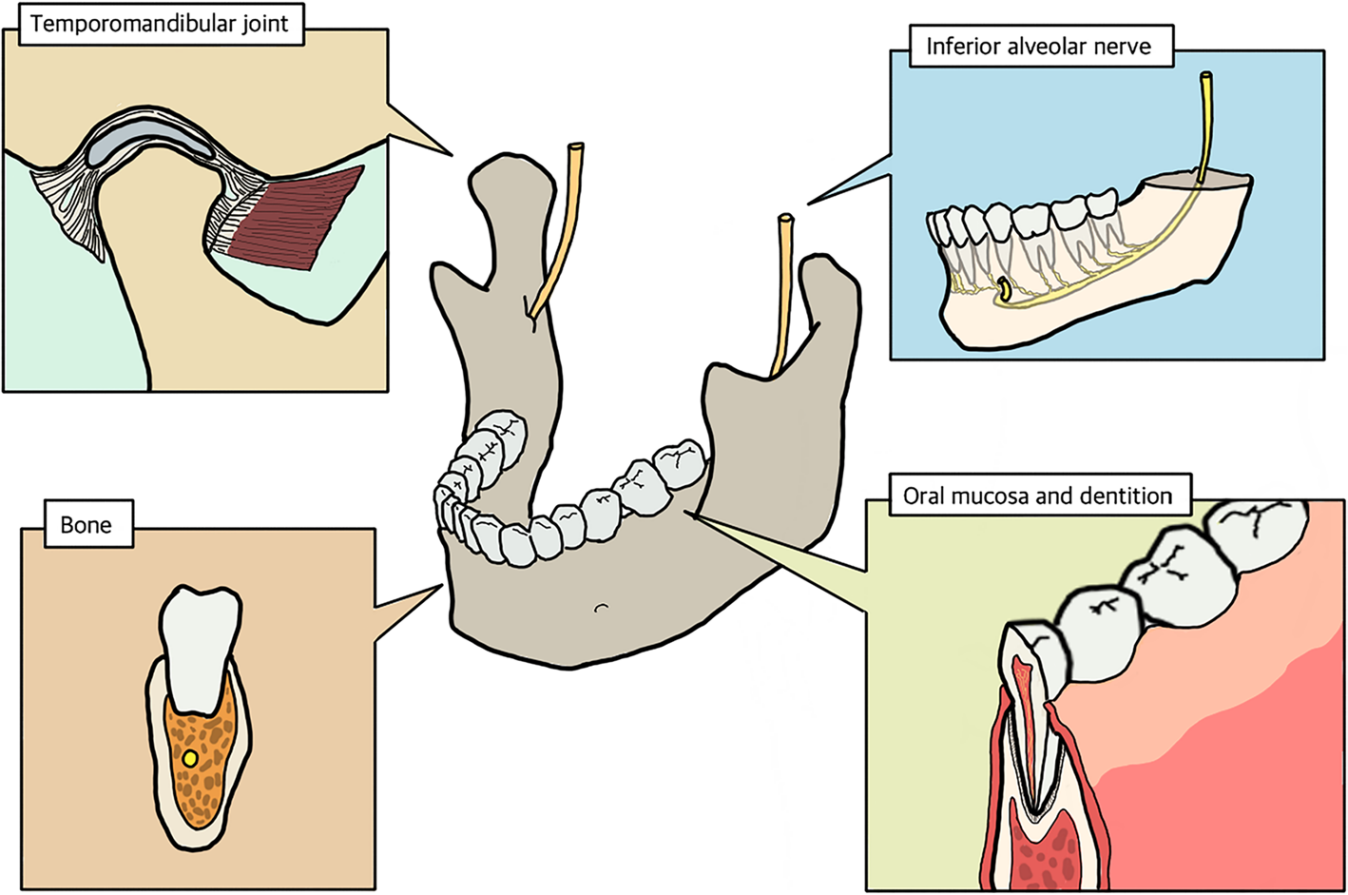
Composite tissue anatomy of mandible, for composite tissue reconstruction of mandible, the anatomic component generally include temporomandibular joint, dentition, oral mucosa, inferior alveolar nerve, and parenchymal bone frame

A mandible is a separated facial bone that is connected to other facial bones with the muscles and ligaments in the maxillofacial area. When considering composite reconstruction of mandibular defects, the anatomical components of the mandible are broken down into temporomandibular joint, dentition, oral mucosa, inferior alveolar nerve, and parenchymal bone frame [11]. Most clinical situations require composite reconstruction with more than two tissue components, especially in case of a segmental defect of the mandible. Therefore, in designing the 3D bio-printing, anatomic components should be considered in terms of composite tissue reconstruction (Fig. 1).

### Temporomandibular joint (TMJ)

The mandible is held to the cranium by temporomandibular joints (TMJs) bilaterally with masticatory muscles and adjunctive ligaments. The articular disc is a specific tissue, composed of fibrocartilage, which is located between the mandibular condyle and condylar fossa of the temporal bone [12]. TMJs control the mouth opening with translation and sliding movements and stabilize the lateral movement of the contralateral side, which require a long-term resistance to masticatory loading for all life cycles [13]. Therefore, composite tissue repair includes the fibrous tissue, bone, cartilage, and mechanical stress becomes critical consideration for the functional reconstruction of the TMJ complex.

### Dentition

Teeth are hard structures implanted in the body of the mandible which has functions of breaking down the food and pronouncing words. Teeth are composed of hard tissue, enamel and dentin, and soft tissue involving pulp core, which is a neurovascular bundle and periodontal ligament [14, 15]. Regeneration of the components in dentition is currently not achievable with 3D bio-printing technology [16]. However, dental implants made of titanium can be a successful alternative to achieve normal dental occlusion. In the reconstruction of the mandible, the regeneration of the dental structure is only considered once adequate reconstruction of the mandibular defect is achieved, and the dental implant is a valid option for the repair of dentition [17]. In general, the mandibular reconstruction mainly focuses on reconstructing the bony tissue of the body of the mandible, which is then used as a platform in which dental prosthesis can be implanted [18, 19].

### Oral mucosa

Most mandibular defects are accompanied by oral mucosa which includes keratinized gingiva and mucosa proper. Composite tissue reconstruction using microvascular osteocutaneous free flap such as fibula free flap and deep circumflex iliac artery free flap need concomitant harvesting of soft tissue to repair defects of the oral mucosa [20–22]. Soft tissue coverage protects reconstructed hard tissue in the defect of the bone and enhances functional stability for mandibular movement. For the long-term success of the dental implant, immobilized soft tissue like keratinized gingiva is necessary for the prevention of food impaction and maintenance of hygiene [23, 24]. Regeneration of keratinized soft tissue with supporting bone should be considered at the initial stage of bio-printing for the mandibular reconstruction.

### Inferior alveolar nerve

An inferior alveolar nerve is the largest branch of the mandibular nerve and passes through the body of the mandible. The nerve enters the lingula of the mandibular ramus and comes out through the mental foramen of the mandibular body, which innervates the gingiva, teeth, periodontal ligament, and skin of the chin area [24, 25]. The occurrence of large defects in the mandible usually involves loss of the inferior alveolar nerve. Sensory nerve regeneration is still a difficult process especially when trying to be accomplished with the reconstruction of the bone tissue. However, proprioception of tooth and gingiva and touch sensory of the facial skin give feedback to reconstructed composite tissue, thereby coordinating a delicate masticatory system [26–28]. Recovery of inferior alveolar nerve function accompanied by regeneration of bone tissue would be a critical factor in improving the quality of comprehensive reconstruction of a composite structure of the mandible [28, 29].

### Mandibular bone

The facial lower third part is supported by the mandibular body and ramus. Mandible develops through intramembranous ossification, consequently composed of sturdy cortical plate and cell-rich bone marrow space [30]. Mandibular bone stabilizes facial soft tissue by presenting attachment of masticatory and facial expression muscles. The alveolar part of the mandible supports dentition, which makes a dental arch for masticatory action. The body of the mandible holds alveolar parts and endures functional stress and protects the airway and lower third of the facial compartment [31]. Therefore, in the design of 3D bio-printing of the mandible, facial contour and mechanical stress from mastication should be considered.

## 3D bio-printing technology

**Fig. 2:**
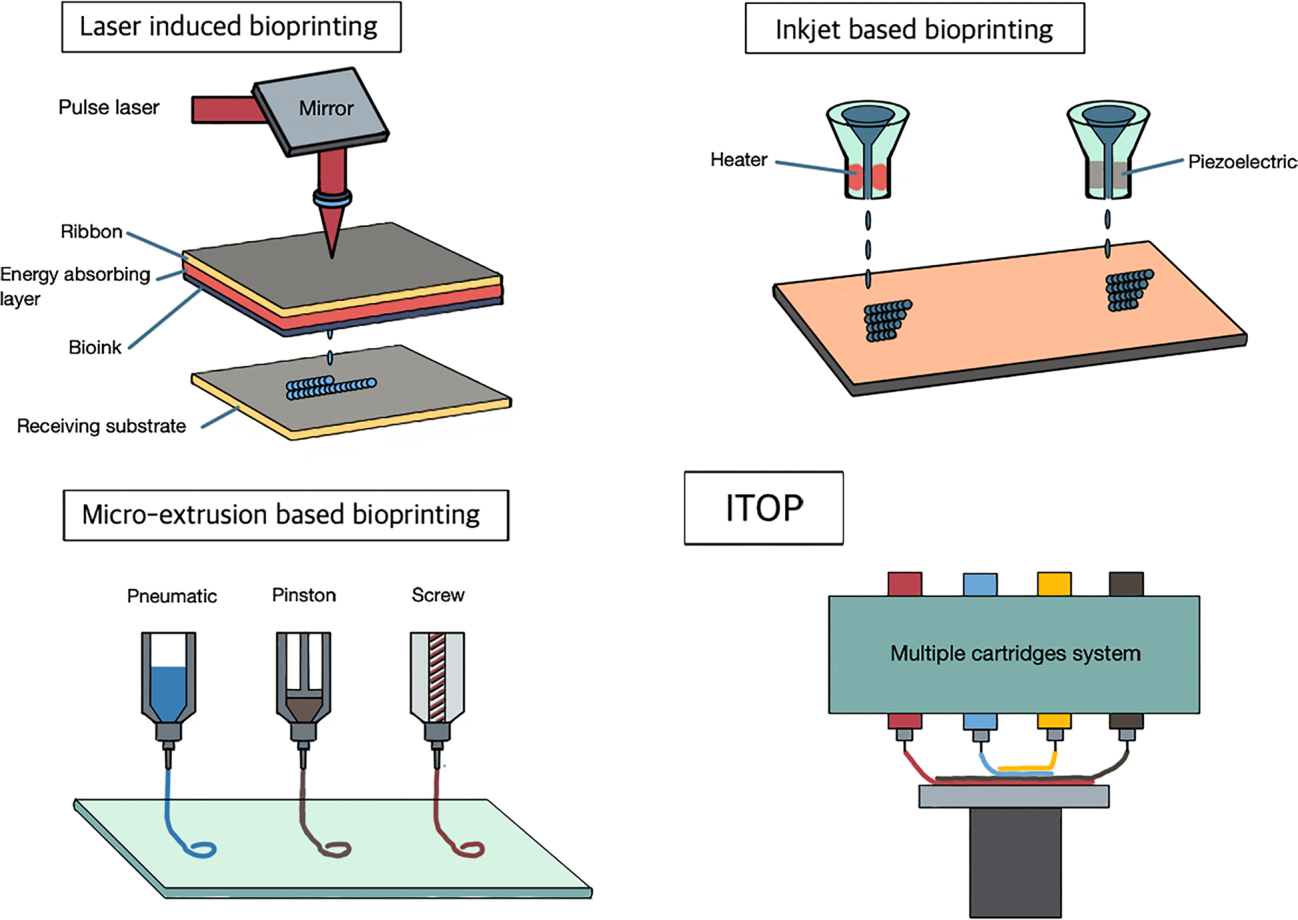
Bio-printing modalities, the primary bio-printing methods include laser-induced bio-printing (LIB), inkjet-based bio-printing (IBB), and micro-extrusion-based bio-printing (EBB) according to their performance modalities. In addition, integrated tissue and organ printing (ITOP) system, equipped with multiple cartridges can print composite tissue at one time

Bio-printing is a performance system actuated by additive manufacturing that controls the printing of biomaterials under precise coordination. It uses computerized dimensions to reconstruct biological defects in the human body [32]. It should print out a stabilized 3D structure which was designed to incarnate original composite tissue based on the medical image without damage to biomaterials. Availability, especially to simultaneously print multiple biomaterials, is critical to perform the 3D bio-printing of composite tissue. The primary bio-printing methods include laser-induced bio-printing (LIB), inkjet-based bio-printing (IBB), and micro-extrusion-based bio-printing (EBB) according to their performance modalities (Fig. 2) [33].

### Laser-induced bio-printing

Laser-induced bio-printing (LIB) consists of a pulse energy laser, ribbon substrate coated with bioink and receiving plate. When laser pulse energy is transferred to the ribbon layer, the energy absorbing layer is then activated, which dispatches bioink onto receiving substrate [34]. In general, LIB can make a relatively deliberate printing structure while maintaining cell viability; thus, this technique would be expected to be applied to single-cell-layered structures such as the oral mucosa, blood vessels, and skin [35]. However, printed products do not provide sufficient mechanical strength to withstand functional loading in the mandibular bone. Accordingly, it is useful for a layer structure which needs simplicity, precision, and pliability [36]. In terms of composite tissue reconstruction of the mandible, it would be suitable for oral mucosa or periosteum.

### Inkjet based bio-printing

Biomaterials usually containing cells and bioactive molecules are transformed to droplets by the piezoelectric or thermal-based energy source when inkjet based bio-printing (IBB) gets actuated [37]. IBB modality needs lower viscosity for flowable printability, which makes it difficult to print complex 3D structures with proper hardness [33, 38]. Therefore, the structures which are delicate but not requiring high strength would be an indication, for example, the muscle, ligament, blood vessel, and cartilage of the ear and nose [34]. Biopaper of calcium chloride solution can be used as a stiffness-inducing background to reinforce structure and strength [39, 40]. Bioreactor additionally would be used for further maturation of printed structures before graft implantation [41, 42]. Even though bone cells can be printed in the IBB modality, IBB would not be a mainstay bio-printing modality for mandible reconstruction especially for bone. Masticatory and contractile forces from facial soft tissue are also hurdles for a successful outcome [34]. IBB modality can be limitedly used for the bio-printing of soft tissue around the mandibular bone.

### Micro-extrusion based bio-printing

Extrusion-based bio-printing (EBB), a kind of fused deposition modeling (FDM) modality, is a primary bio-printing modality for bone regeneration [34]. Biomaterial in the EBB setting is loaded in the syringe combined with a microscale nozzle. Biomaterials in the syringe are pushed by the pressure of a pneumatic, piston, or screw [43]. Multiple cartridge settings can be devised in EBB, which makes simultaneous printing of various cells and bioactive materials on demand [44]. EBB cannot print biomaterial structures with high precise resolution as LIB or IBB does though, polymers that reinforce mechanical strength can be printed with three-dimensional structures via EBB [32]. In general, the hydrogel is the mainstay media for cells and bioactive molecules that control osteogenic cell ingrowth and vascularization into the implanted scaffold for mandibular reconstruction. However, single hydrogel media are not appropriate for mandibular reconstruction, for it cannot provide sufficient mechanical strength [45]. Therefore, additional biomaterials to complement mechanical strength need to be printed at the same time. EBB can be designed to print composite tissue by setting multiple biomaterial cartridges, which make mandibular regeneration affordable [32, 44].

Recently, an integrated tissue and organ printing (ITOP) system, equipped with multiple cartridges can print out polymers which reinforce the printed structure as well as hydrogel. For this reason, ITOP overcomes the limitation of mechanical strength. In terms of mandibular reconstruction, the printed materials should continuously withstand masticatory stress and needs to support the facial muscle and skin; thus, ITOP would be an absolutely suitable indication (Fig. 2) [44].

## Biomaterials for reconstruction

Reconstructed mandibles should bear masticatory stress and contractile force from the facial muscle and skin. On the other hand, regenerative materials with over stiffness inflict significant strain on the overlying skin that consequently incurs skin dehiscence. Therefore, the materials for bio-printing should have mechanical strength, resilience, and biocompatibility [46, 47]. Scaffolds, cells, and bioactive molecules are essential components to meet the required conditions for successful reconstruction [48]. Various materials have been suggested to be utilized for 3D printing of mandible reconstruction. Simple scaffolds of single materials can be used for reconstruction, which would be simply designed with low effectiveness and applied promptly to the operation room. However, additional cells and bioactive molecules can be combined for the improvement of performance in the human body (Table 1) [34, 49].

**Table 1:** Biomaterials for composite reconstruction of mandible

Biomaterials	Components	Features
**Scaffold**	Bioceramic α-TCP β-TCP hydroxyapatite (HA)	Excellent osteoconductivity for bone regeneration, but limited in large defect due to brittleness
	Synthetic polymer PCL (polycaprolactone) PLA (polylactic acid) PLGA (polylactic glycolic acid) PEG (polyethylene glycol)	Favorable mechanical forces and biocompatibility
	Natural polymer Chitosan Agarose Alginate Silk	High biocompatibility and degradability, loading bioactive molecule, poor mechanical strength
**Cells**	Adult stem cell (ASC) Embryonic stem cell-induced pluripotential stem cell (iPSC)	Potential to be differentiated into multiple composite tissue of mandible
**Bioactive** **molecules**	Growth factor (GF) Other cytokines and peptides	Modulating osteogenesis and angiogenesis Potentials of cell mobilization and growth

### Scaffolds

In mandibular reconstruction, scaffold generally comes to be focused on the regeneration of the bone tissue. Utilized materials, composition techniques, and fabrication methods can affect the nature of the scaffold [50]. When the reconstructive materials get implanted into the mandibular defect, the scaffold should resist against infection and provide space with proper mechanical strength as well as guide cell growth and vascularization [1, 51]. The mandibular bone is largely composed of inorganic components (hydroxyapatite) related to hardness and organic components (collagen) related to resilience [52]. Accordingly, several scaffold materials are contemplated, including bioceramics of calcium phosphate components such as α-tricalcium phosphate (TCP), β-TCP, and hydroxyapatite (HA) [53, 54]; biopolymers which are synthetic polymer of PCL (polycaprolactone), PLA (polylactic acid), PLGA (polylactic glycolic acid), and PEG (polyethylene glycol) [55–59] and natural polymer of chitosan, agarose, alginate, and silk [60–62].

PCL has a slower speed of degradation in the human body; on the other hand, it can withstand relatively large force and maintain the space [63, 64]. PLA, PLGA, and PEG show rapid degradation while having favorable biocompatibility compared to PCL [65]. Natural polymers have higher biocompatibililty and rapid degradation in addition to being available to deliver bioactive molecules, therefore are disadvantageous in terms of mechanical stress [66]. Bioceramics has excellent osteoconductivity, useful for the regeneration of the mandibular bone. However, their brittleness limits application for large defects [67, 68], the scaffold for mandible reconstruction, especially having complex anatomic structures including curvature, condyle, and needs combination of bioceramics, synthetic, and natural polymers that can complement their defects.

### Cells

Reconstructive mandibles are implanted in the defect, from which osteogenesis and angiogenesis will ensue. Delayed cell ingrowth endangers the successful integration of reconstructive materials due to the following infection. For effective reconstruction, specific cell lineage needs to be integrated onto scaffolds. There are three types of available stem cells that can be considered in mandibular reconstruction; adult stem cells (ASCs), embryonic stem cells, and induced pluripotential stem cells (iPSCs) [48]. ASCs are a popular source of mesenchymal stem cells (MSCs) which is a mainstay for mandibular reconstruction, because they are easily collectible and cost-effective [69]. MSC can be harvested from the matured bone marrow and adipose, then differentiated into various mesenchymal origin tissues which are the bone, cartilage, periodontal ligament, dental pulp, developing teeth, and gingival epithelium [70, 71]. This is an advantageous potential for composite tissue reconstruction of the mandible.

### Bioactive molecule

Extracellular matrix (ECM) is a medium to provide a microenvironment for the biologic interaction of reconstructive materials with the recipient bed [72, 73]. Bioink, simulating natural ECM, is a synthetic hydrogel background, which includes collagen and gelatin methacrylate (GelMA) [32]. Hydrogel can be engineered to contain growth factors including transforming growth factor-β (TGF-β), fibroblast growth factor (FGF), vascular endothelial growth factor (VEGF), platelet-derived growth factor (PDGF), insulin-like growth factor (IGF), and bone morphogenic proteins (BMPs) that help regenerative action in the human body as well as cells [32, 38]. Bone regeneration with BMP-2 can produce a comparable amount of bone formation to the autogenous graft, and it reduce some post-surgical complications, operation time, and hospitalization duration [74]. Delivered growth factors control MSC to modulate bone formation, angiogenesis, and newly growth of other mesenchymal origin tissue [75–77]. Cytokines and modified peptides that have potentials for cell mobilization and growth can be conjugated to hydrogel to be delivered as a form of bio reconstructive material with stability [78–80]. At last, nanotechnology can enhance the ability to mimic native bone ECM and improve the bone regeneration process [81].

## Factors for clinical application

Mandible defects caused by trauma, osteomyelitis, and head and neck cancer require multiple composite tissues such as oral mucosa, dentition, and bone [82]. Adequate bone volume and vertical height of reconstructed bone are important for further surgical planning and as an indicator of the quality of reconstruction [8]. Reconstructed mandibles should provide the function of mastication and speak as well as support for facial soft tissue and airway, coping with harsh conditions of oral microflora and life-long masticatory stress and continuous force from adjacent facial soft tissue [83, 84]. Therefore, for successful mandible reconstruction with long-term stability, critical factors should be included at the stage of designing the reconstruction printing. Some of the considerations include mechanical strength, infection resistance, biocompatibility, and future rehabilitation [85].

### Mechanical strength

3D printed biomaterials for mandible reconstruction come to confront the challenge to maintain the functional space from the mechanical stress during while functioning and tensional force of facial soft tissue as soon as they are set into the defect [83, 86]. These stresses should not exceed the yield point, which can be measured from compression, bending, or tensile force [87]. Reinforced titanium plate has been used for decades though; accumulated clinical results are not satisfactory especially in the cases of long-term function and postoperative radiation therapy. They exhibited screw loosening, fracture of titanium plates, and exposure of fixation materials out of the skin or oral mucosa which would eventually ensue infection requiring complete removal of reconstructive materials [88]. Moreover, the future oral rehabilitation using dental implant usually became limited, for reinforced titanium plates were not able to provide the oral prosthesis with a supporting hard tissue table for rehabilitation of occlusion [89, 90]. A titanium mesh structure was also suggested that presented an occlusal table for an oral prosthesis as designed with a similar dimension to the original mandible. However, titanium still risks the potential of exposure and breakage of material entailing infection regardless of design [82]. For this reason, Jeong et al. reported that the bioabsorbable scaffold with virtual simulation and 3D printing techniques may replace traditional non-absorbable implants in the future by virtue of its accuracy and biocompatible properties [91].

Osteocutaneous free flap (OCFF) is the gold standard for the composite tissue reconstruction of the mandible as of now. OCFF is the most biocompatible reconstructive option, which has the similar mechanical strength to the original mandible as well as long-term stability due to its biologic integration with the recipient bed after surgery [92–94]. However, OCFF entails additional surgery to harvest composite tissue that requires patients’ longer operation time and healing period with second operation wound. Consequently, 3D bio-printing for mandible reconstruction should aim to overcome the limitation of OCFFs, while taking their advantages.

### Expeditious vascularization

Successful integrations of reconstruction materials thoroughly depend on preventing postoperative infection. In terms of the environment at mandible defect, infection would be a constantly existing menace [95]. The oral cavity has influential factors that are able to make an infectious source at any time which are oral microflora and food impaction. When patients start mastication, oral mucosa that was sutured after reconstructive surgery would be exposed to mechanical irritants of food stuff [96, 97]. In the case of OCFF, oral mucosa gets positioned on the vascularized free flap that can make blood supply to overlying mucosa and even present well-vascularized soft tissue covering the bone tissue, thereby protecting underlying reconstructed mandible until the OCFF can be successfully integrated into the defect [22, 98]. However, the graft materials that failed to get early vascularization would easily be exposed out of the oral cavity and get an infection by oral microflora, which leads to failure of reconstruction [24, 82].

The disparity from defect sizes determines reconstructive options. When the mandibular defect is confined to the alveolar bone level that has underlying the basal bone, the graft can simply gain the blood supply from the adjacent bone marrow. While in segmental bone defects, the graft risks early exposure and subsequent infection if it could not promptly be vascularized. In the defect from 4 to 6 cm in length of the mandibular segment, an autogenous bone graft basically should be considered as a first coming option. Moreover, for longer than 6 cm defects, OCFF would be an inevitable treatment strategy as of now [99, 100]. The limitation of 3D printed biomaterial corresponds to the long segmental dimension that has been addressed by OCFF; thus, those require prompt vascularization to avoid fatal infection. In order to reduce surgical site infection, stable soft tissue coverage over bone graft is essential. For this reason, it is highly recommended to harvest skin pedicles with bone graft. At the step of designing biomaterials, reconstructive mandibles should be considered to be concomitant with oral mucosa like composite tissue and vessel-inducing bioactive molecules or cells to achieve expeditious vascularization that protects graft materials.

### Rehabilitation of occlusion

Most cases of mandibular defect accompany the loss of multiple dentitions, which consequently causes an occlusal collision. Rehabilitation of occlusion is the endpoint of mandibular reconstruction [82, 101]. Composite tissue reconstruction of the mandible usually does not mean the simultaneous restoration of dentition, even in reconstruction with OCFF. Although some OCFF case reports suggested simultaneous dental implant installation, the indication seems to be still limited [9, 102]. Prolonged surgical time and ischemic time for immediate implant placement may impact flap survival and can increase a risk for direct injury to the vessel pedicle [103]. Occlusal rehabilitation generally can be planned when the long-term stability of reconstruction is biologically confirmed in patients’ body. Even in reconstruction with OCFF which is the most biocompatible option, rehabilitation using dental implants is a challenging procedure. Fibula free flap (FFF) which is one of the most popular OCFF options can only have about 1-cm bone height which is not enough for an ideal crown-to-root ratio in terms of dental prosthesis [101, 104, 105]. Deep circumflex iliac artery free flap (DICAFF) can provide the large quantity of bone dimension for both immediate and delayed placement of dental implants [106, 107]. Compared with FFF, it is limited to relatively short segmental defects of the mandible due to its short pedicle length [106, 107]. In addition, OCFFs are not able to provide ideal peri-implant tissue such as keratinized gingiva [108]. Therefore, 3D printed biomaterials for mandibular reconstruction need a sufficient bone table to accommodate dental implants and keratinized epithelium to provide healthy peri-implant soft tissue.

## On-demand process for reconstruction

As mandibular defects appear in various morphologies which could be body, condyle, full dentition, or a combination of them, the design of 3D bio-printing should be customized according to the cases [34, 109]. Therefore, a comprehensive system of flow process based on on-demand for individual patients needed to be established, which can integrate the anatomic image of individual patients, decide the composition of biomaterial, design of the reconstructive material, and get delivered to the operation field [7].

**Fig. 3:**
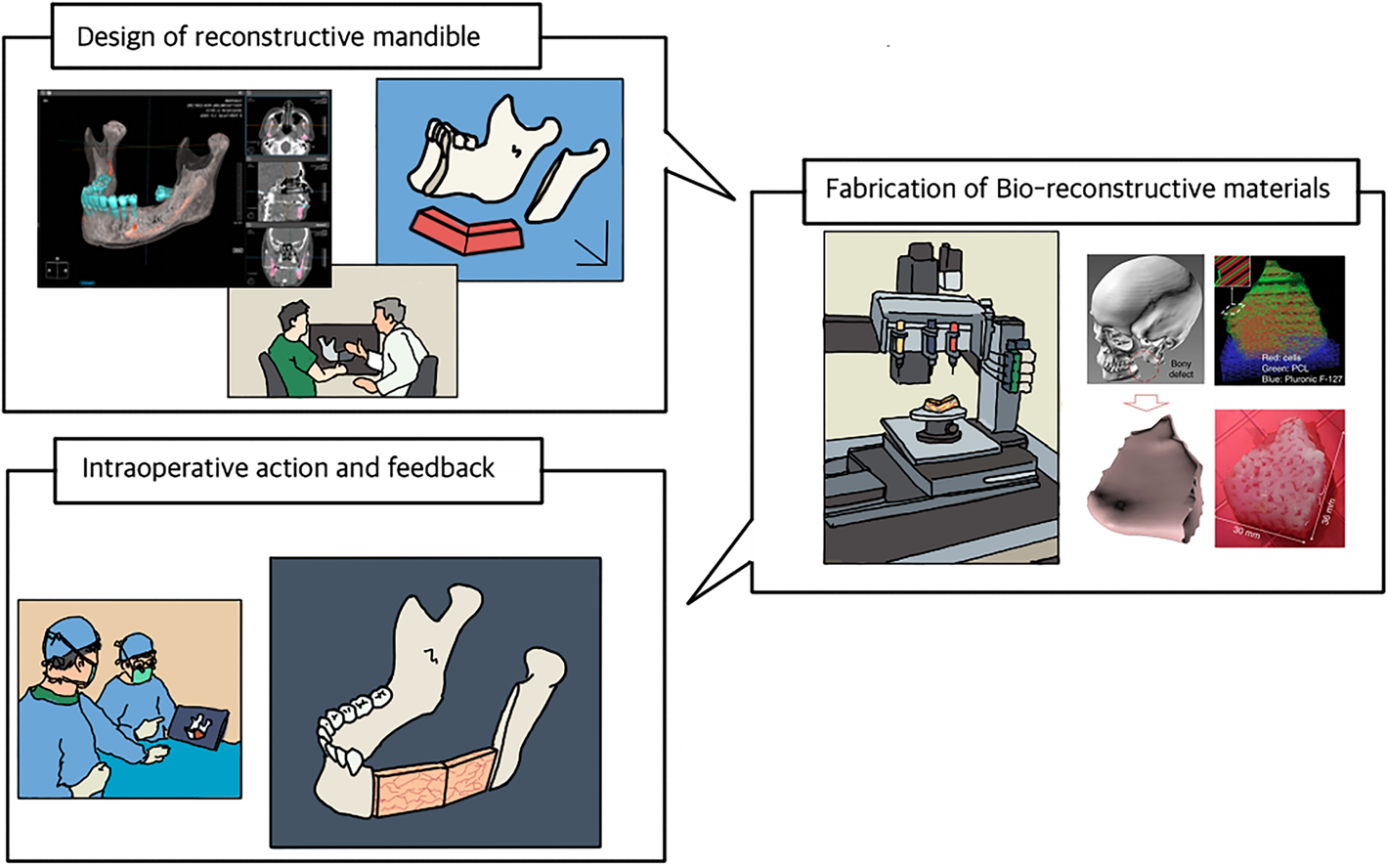
On-demand process for customized mandible reconstruction, the comprehensive on-demand system for individual patients needed to be established, which can integrate anatomic image of individual patients, decide composition of biomaterial, design of reconstructive material, and get delivered to operation field for successful mandibular reconstruction (some part of this figure was with permission from Springer Nature. Copyright 2016)

The on-demand system should be an online platform on which all members of the reconstruction team including surgeons, researchers, engineers, and radiologists can easily access and communicate regarding an uploaded case, then devise surgical templates according to the treatment plan and design reconstructive mandible with scaffold, cells, and bioactive materials that are required as patient’s own case (Fig. 3).

### Design of neo-mandible

Conventional panoramic radiograph is insufficient to analyze the anterior-posterior dimension and the influence of free flap rotation on the rehabilitation of patients [110]. Multiple image modality is essential to evaluate the exact volume required for mandibular reconstruction [8].

Multiple image modalities such as CT, MRI, and PET CT can be used for diagnosis and preliminary planning for reconstructive surgery. Multiple modalities would be helpful to figure out and customize the cases [32, 48]. CT has been a critical image modality for the reconstruction of maxillofacial defects. Because it can make 3D reconstruction images that present detailed anatomy of individual cases and precise spatial information for 3D printing compared to other modalities [111, 112].

After acquiring CT images followed by case analysis, virtual surgery is performed on on-demand system by a surgeon; herein, the decision for the dimension of reconstructive material is confirmed. The feasible composition of biomaterial including scaffold, cells, and bioactive molecules is discussed at this stage according to a three-dimensional design by team members.

In addition, a surgical guide should be designed concomitant with bio-printing material. The surgical guide is a template that helps guide 3D printed bioreconstructive material at planned position intraoperatively. Based on acquired 3D CT images, the surgical guide is designed to reproduce the surgical planning to put the reconstructive materials in the defect with precision and then printed to be used intraoperatively with CAD-CAM technology [7, 10, 85, 110].

### Bio-fibrication

Modifications of design through multidisciplinary specialists on an online platform will be processed repeatedly until the most applicable design would be set up for a forthcoming surgery. After the final design is confirmed, the composite tissue can be printed as multiple cartridge manners such as ITOP [44]. Selected materials for scaffold, cells, and bioactive molecules for the individual case are loaded on the 3D bioprinter and simultaneously printed according to the shape and dimension which were determined at the planning stage. Completed reconstructive material then will be confirmed by on-demand platform, either.

### Intraoperative feedback

The printed products are then moved to the operation room for reconstructive surgery. Bone defect preparation as the recipient bed is preceded before setting the reconstructive material as helped by a surgical guide. The 3D-printed surgical guide gives information for the accurate setting of reconstructive material [113–115]. During the operation, a surgeon can check the procedure goes as it was planned intraoperatively with a tablet PC interconnected to an online platform. All data from intraoperative procedures and postoperative results were stored in the database of online platforms as feedback. The database would be conducive to the improvement of the design in reconstructive material for the next coming cases.

## Conclusion

Mandible reconstruction based on 3D bio-printing needs coordination with oral mucosa, dentition, and other facial bones and muscles; thus, critical factors including mechanical strength, facial contour, mastication, and maintenance of airway should be contemplated at the initial stage of designing biomaterial.

Harsh environments in maxillofacial areas, such as continuous mechanical stress, salivary fistula and radiation therapy, risks of breakage, and exposure to implanted grafts may cause fatal infection and failure of reconstruction. Biomaterials, basically composed of scaffolds, cells and bioactive molecules, require sufficient mechanical strength and biocompatibility to cope with disadvantageous conditions. OCFF is still the gold standard for composite tissue reconstruction of mandibles, even though it requires additional flap harvesting surgery and probable donor site morbidity. As of now, 3D bio-printing technology seems to be not feasible in case of a complete segmental defect of the mandible. In the near future, the ITOP bio-printer will be able to print out multiple tissues and is expected to be used in the 3D composite reconstruction of the mandible to take over the place of OCFF.

3D CAD-CAM became an essential technology for the forthcoming 3D bio-printing reconstruction of mandibles. The design of mandibular reconstruction with biomaterial should be customized according to dimension and tissue components in individual patients’ mandibular defects. For surgical templates, non-biologic 3D printing has already been utilized based on CAD-CAM technology. Through on-demand platform, multidisciplinary approach to the reconstruction can be performed for designing a surgical template and reconstructive mandible based on acquired medical image. Then, printing surgical template and reconstructive mandible will be done. Although the development of 3D bio-printing for composite mandibular reconstruction is on the way as of now, on-demand platform for comprehensive reconstructive treatment should be set up accordingly for forthcoming technological advances.

## Data Availability

Not applicable

## Abbreviations

ASCs: Adult stem cells
BMPs: Bone morphogenic proteins
CAD-CAM: Computer-aided design/computer-aided manufacturing
CT: Computed tomography
DICAFF: Deep circumflex iliac artery free flap
EBB: Micro-extrusion-based bio-printing
ECM: Extracellular matrix
FDM: Fused deposition modeling
FGF: Fibroblast growth factor
FFF: Fibula free flap
GelMA: Gelatin methacrylate
HA: Hydroxyapatite
IBB: Inkjet-based bio-printing
IGF: Insulin-like growth factor
iPSCs: Embryonic stem cells and induced pluripotential stem cells
ITOP: Integrated tissue and organ printing
LIB: Laser-induced bio-printing
MRI: Magnetic resonance imaging
MSCs: Mesenchymal stem cells
PCL: Polycaprolactone
PDGF: Platelet-derived growth factor
PEG: Polyethylene glycol
PET CT: Positron emission tomography-computed tomography
PLA: Polylactic acid
PLGA: Polylactic glycolic acid
OCFF: Osteocutaneous-vascularized free flap
TCP: Tricalcium phosphate
TGF: Transforming growth factor
TMJs: Temporomandibular joints
VEGF: Vascular endothelial growth factor
